# Effects of weight training on cognitive functions in elderly with Alzheimer's disease

**DOI:** 10.1590/S1980-57642012DN06040009

**Published:** 2012

**Authors:** Thays Martins Vital, Salma S. Soleman Hernández, Renata Valle Pedroso, Camila Vieira Ligo Teixeira, Marcelo Garuffi, Angelica Miki Stein, José Luiz Riani Costa, Florindo Stella

**Affiliations:** 1 Institute of Biosciences, Universidade Estadual Paulista (UNESP), Physical Activity and Aging Lab (LAFE), Rio Claro SP, Brazil.; 2 Geriatric Psychiatry Clinic, Universidade Estadual de Campinas (UNICAMP), Campinas SP, Brazil.

**Keywords:** cognition, Alzheimer's disease, resistance training

## Abstract

Deterioration in cognitive functions is characteristic in Alzheimer's disease (AD) and may be associated with decline in daily living activities with consequent reduced quality of life.

**Objective:**

To analyze weight training effects on cognitive functions in elderly with AD.

**Subjects:**

34 elderly with AD were allocated into two groups: Training Group (TG) and Social Gathering Group (SGG).

**Methods:**

Global cognitive status was determined using the Mini-Mental State Exam. Specific cognitive functions were measured using the Brief Cognitive Battery, Clock Drawing Test and Verbal Fluency Test. The protocols were performed three times a week, one hour per session. The weight training protocol consisted of three sets of 20 repetitions, with two minutes of rest between sets and exercises. The activities proposed for the SGG were not systematized and aimed at promoting social interaction among patients. The statistical analyses were performed with the U Mann Whitney and Wilcoxon tests for group comparisons. All analyses were considered statistically significant at a p-value of 0.05.

**Results:**

There were no significant differences associated to the effects of the practice of weight training on cognition in AD patients.

**Conclusion:**

In this study, no improvement in cognitive functions was evident in elderly with AD who followed a low intensity resistance exercise protocol. Thus, future studies could evaluate the effect of more intense exercise programs.

## Abstract (pt)

A deterioração das funções cognitivas é característica na doença de Alzheimer (DA) e se relaciona com uma pior realização das atividades de vida diária levando a uma pior qualidade de vida.

**Objetivo:**

Analisar os efeitos de um programa de treinamento com pesos nas funções cognitivas de idosos com DA.

**Sujeitos:**

34 idosos com DA foram alocados em dois grupos: Grupo Treinamento (GT) e Grupo de Convívio Social (GCS).

**Métodos:**

Para caracterização do perfil cognitivo global utilizou-se o Mini-Exame de Estado Mental. Para a mensuração das funções cognitivas foram utilizadas: Bateria Cognitiva Breve, Teste do Desenho do Relógio e teste de Fluência Verbal. Os protocolos oferecidos foram realizados três vezes na semana, durante uma hora. O protocolo de treinamento do GT consistiu em realizar três séries de 20 repetições para cada exercício, com dois minutos de intervalo entre séries e entre exercícios. As atividades propostas para o GCS não foram sistematizadas e tiveram por objetivo promover uma socialização dos pacientes. A análise estatística consistiu na utilização dos testes U Mann Whitney e Wilcoxon para comparação intra e entre grupos. Adotou-se nível de significância de 5% para todas as análises.

**Resultados:**

Não foram evidenciadas diferenças significativas relacionadas aos efeitos da prática do treinamento com pesos na cognição de idosos com DA.

**Conclusão:**

Neste estudo não houve melhora nas funções cognitivas em idosos com DA que realizaram um protocolo de exercícios resistidos de intensidade leve. Assim, estudos futuros poderiam verificar o efeito de exercícios mais intensos.

## INTRODUCTION

Alzheimer's disease (AD) is characterized by progressive and irreversible cognitive decline resulting from neuronal apoptosis, predominantly caused by excessive accumulation of neurofibrillary tangles and beta-amyloid brain plaques.^1-4^

The key characteristic for clinical diagnosis of AD is memory impairment and at least one other disorder such as apraxia, agnosia and aphasia that compromise the professional and social life of the individual.^5^

Changes in cognitive functions, a characteristic of AD, are associated with impairment in instrumental and basic activities of daily living.^6,7^ These modifications can affect the quality of life of patients and caregivers as well as increase the chance of early institutionalization of this patient group.^8-11^

Some studies have shown that physical activity in older adults with AD can contribute positively by attenuating decline in cognitive function.^12-15^ The possible mechanisms of physical exercise in improving cognition include an increase in cerebral blood flow, generating a greater supply of oxygen and other energy substrates, changes in the synthesis and use of important neurotransmitters for brain activity and also positive interference in brain angiogenesis, synaptogenesis and neurogenesis.^14,16-19^

Most studies involving physical activity and AD have used general or aerobic training protocols.^20-26^ Few studies however, have investigated the effects of weight training on cognitive functions in patients with AD.

Studies with cognitively preserved elderly have shown that weight training produces significant gains in cognitive functions.^27-30^

## OBJECTIVE

To analyze the effects of a weight training program on cognitive functions in elderly with Alzheimer's disease.

## METHODS

**Subjects.** All participants in this study were part of the of *Programa de Cinesioterapia Funcional e Cognitiva em idosos com doença de Alzheimer* (PRO-CDA),^31^ an extension project offered by the Department of Physical Education, Institute of Biosciences, Universidade Estadual Paulista, Rio Claro campus.

The elderly entered the program through medical indications, and also by their own and/or caregiver's initiative after the announcement of the project in print and broadcast media.

The sample comprised a total of 34 elderly with mild-to-moderate stage of disease according to scores on the Clinical Dementia Rating scale.^32,33^

The study inclusion criteria were: clinical diagnosis of AD, according to the criteria of DSM-IV-TR^5^ applied by specialists in the area, and elderly with AD of mild and moderate severities.

In order to characterize the level of physical activity of the participants, the Modified Baecke Questionnaire for Elderly was applied.^34^

The sample was divided into the two groups by convenience, adopting a similar distribution in terms of age, sex, education and clinical condition (variables characterizing the sample). Thus, the Training Group (TG) consisted of 17 elderly and the Social Gathering Group (SGG) also comprised 17 elderly. The evaluation procedure described below was adopted for both groups.

Caregivers for the elderly signed the consent form, approved along with the research project by the Research Ethics Committee of the Universidade Estadual Paulista, under protocol number 4826.

**Evaluation procedures.** Firstly, all elderly underwent diagnostic confirmation with a psychiatrist, specialized in Geriatric Psychiatry. After confirming the diagnosis for those patients that met the inclusion criteria of the study, a second evaluation was scheduled with a blinded evaluator.

Subsequently, the first evaluations were scheduled, prior to the familiarization period of this study, in a single visit lasting approximately 1 hour during which the elderly were evaluated using the tests described below. All evaluations were performed during the afternoon in a quiet environment.

The Mini-Mental State Examination (MMSE) was employed to characterize global cognitive status.^35^

As the MMSE is influenced by education, reference values were proposed in order to distinguish subjects with possible cognitive deficits. Brucki et al.^36^ analyzed a Brazilian sample and suggested the following values ​or studies in this area: illiterates, 20 points; subjects with 1-4 years of schooling, 25 points, 5-8 years, 26,5; 9-11 years, 28 and for individuals with over 11 years of education, 29 points.

The Brief Cognitive Battery (BCB)^37,38^ is a screening instrument that measures cognitive processes, especially memory. This instrument has been used to identify cognitive changes and is divided into domains namely: identification, incidental memory, immediate memory, learning, delayed recall and recognition. Importantly, this instrument is not influenced by education.^39^

This Battery entails presenting the subject with 10 common figures (shoe, spoon, comb, tree, turtle, key, airplane, house, book and bucket). These objects must first be named by the subject (identification/appointment), who are then immediately asked to evoke each item, without having been informed that the objects were to be memorized (incidental memory). In the next stage, the figures are shown again and the subject is required to memorize them (immediate memory), and the procedure is repeated (learning). Again, the figures are presented to the subject, with instructions to memorize them and recall them later, after 5 minutes. However, before this evocation, the examiner introduces two tests that serve as distracters: the Verbal Fluency Test and Clock Drawing Test (described below). After these two tests, the subject is asked to evoke the figures previously memorized (5 minutes). Finally, the 10 figures are presented along with 10 others and the participant has to recognize the figures originally presented (recognition). The total score is given as number of successes in each domain, except for the recognition domain in which the number of intrusions (errors) must be subtracted from the number of successes.

The Clock Drawing Test^40^ consists of the task of drawing a clock, inserting pointers and numbers, and setting it to a given hour (e.g. 2:45 a.m.). This test assesses executive functions (planning, abstract thinking, logical and monitoring of executive processing).

The Verbal Fluency test^41^ animals category is a simple test characterized by the ability of the subject to name as many animals as possible within one minute. This test measures processing speed, semantic memory and especially language.

All evaluations described above were applied pre and post intervention.

**Design and intervention protocol**. The study has a longitudinal design, with an intervention period of 16 weeks. Both intervention protocols involved sessions held three times a week on nonconsecutive days, with each sessions lasting one hour. All elderly had a minimum attendance rate of 70% in their respective protocols.

**Weight training experimental protocol - Training Group (TG)**. The weight training protocol followed an alternating order of segments, first lower then upper limbs with five exercises performed, always starting with the large muscle groups.

Initially, there was a familiarization period of the patients in the training room (gym) involving three sessions held on nonconsecutive days, during which patients performed one set of 20 repetitions for each exercise, with minimal load. This period preceded the period of the experimental protocol and was not included in the 16 weeks of training.

The exercises selected for the implementation of this protocol were: the Peck Deck, the Pull Down, Leg Press, supported barbell curls performed with free weights and Triceps Pulley. Participants were encouraged to maintain the same movement speed during concentric and eccentric contractions of approximately two seconds.^42^

*Warming up* - Immediately before the exercise sessions, there was a warm-up comprising a set of 20 repetitions with the minimum load for the respective equipment. These loads were approximately 5kg (Peck Deck), 5kg (Pull Down), 7kg (Leg Press), 1kg (Barbell Curls) and 5kg (Triceps pulley).^42^

*Determining training load and overload* - To determine the initial load and subsequent adjustments, a test was performed with two sets of 20 repetitions and a third set until fatigue (participant was instructed to perform as many repetitions as possible), adopting a five-minute interval between attempts. When the last series exceeded 22 repetitions the load was increased. Other parameters such as decrease in speed of execution and voluntary interruption of the movement were used as indicators of fatigue during the determination of load. Both determination and adjustment of loads were performed during the training sessions and were counted in the total volume of training.

The load related to exercising to fatigue in the third set included three sets of 20 repetitions maximum.

Load adjustments were made every 15 days. Adjustment determination was carried out over the course of three sessions, corresponding to one week. Thus, out of the 16 weeks of training, five were dedicated to adjusting load.^42^

*Training sessions* - Immediately after the tests were conducted to determine or adjust load, training sessions were held, which consisted of performing the same exercises at 85% of the load established previously in the determination or adjustment for each exercise.

A two-minute interval was allowed between sets and exercises. There was no stretching, before or after the weight training protocol.

For safety, blood pressure was measured before and immediately after the end of each training session, and also medical and pharmacological accompaniment of patients was not interrupted.^42^

**Experimental protocol - Social Gathering Group (SGG)**. The activities proposed for this group were not systematic, however they were designed to promote socialization of patients and adaptation to the routine of the activities developed in the project. The sessions were conducted in a quiet environment without external influence. Activities such as group dynamics, relaxation, short walks, reading, poetry, musical activities, painting, movies, mimes and recreational activities were carried out.

The lessons were developed by a multidisciplinary team composed of Physiotherapy, Psychology and Physical Education professionals, as well as undergraduate trainees from the university and other private universities in the city.

For activities and materials, colored pencils and felt pens were used for the drawing and painting activities, rubber balls and balloons for group dynamics, and blunt scissors for cutting out activities.^42^

**Statistical analyses.** The U Mann Whitney and Wilcoxon tests were used for all variables analyzed: BCB domains (identification, incidental memory, immediate memory, learning, delayed recall and recognition) and distracter tests (Clock Drawing Test and Verbal Fluency). Descriptive statistics were employed to illustrate the data analyzed. The significance level adopted was 5% for all analyses.

## RESULTS

On the initial analysis, there were no significant differences between groups at pre and post experimental period and likewise no significant differences were found for all intra-group variables (domains, Clock Drawing Test, Verbal Fluency and characteristic variables of the sample).

Table 1 shows the characteristics of the study participants.

**Table 1:** Means and standard deviations of variables characterizing the Training Group (TG) and Social Gathering Group (SGG).

	TG	SGG
Age (years)	78.2±7.3	77.6±6.5
Education (years)	5.4±3.6	5.2±3.8
Mini-Mental State Examination (points)	18.4±4.3	17.7±5.3
Physical activity level (points)	5.4±2.7	3.7±3.8
Duration of disease (months)	31.2±24.7	32.5±39.3
Female	14	13
Male	3	4

Tables 2 and 3 contain the descriptive statistics of the data analyzed by domains of the Brief Cognitive Battery (BCB), Clock Drawing Test (CDT) and Verbal Fluency (VF).

**Table 2:** Medians, range and p-value of domains on BCB for Training Group (TG) and Social Gathering Group (SGG) pre and post intervention.

Domains		TG			SGG		p
		**Range**			**Range**		
		**Median**	**Min. - Max.**		**Median**	**Min. - Max.**	
Identification	Pre	10.0	0-10		10.0	3-10	0.7
	Post	10.0	3-10		10.0	2-10	0.6
Incidental Memory	Pre	2.0	0-7		2.0	0-9	0.6
	Post	2.0	0-9		3.0	0-8	0.9
Immediate Memory	Pre	3.0	1-9		4.0	0-9	0.2
	Post	5.0	0-9		3.0	0-10	0.1
Learning	Pre	4.0	1-8		4.0	0-10	0.9
	Post	5.0	0-10		5.0	0-8	0.7
Delayed Recall	Pre	1.0	0-7		0.0	0-10	0.6
	Post	0.0	0-10		0.0	0-8	0.9
Recognition	Pre	4.0	0-10		5.0	0-10	0.7
	Post	5.0	0-10		5.0	1-10	0.4

**Table 3:** Medians, range and p-value of Clock Drawing Test (CDT) and Verbal Fluency (VF) for Training Group (TG) and Social Gathering Group (SGG) pre and post intervention.

Tests		TG			SGG		p
		**Range**			**Range**		
		**Median**	**Min. - Max.**		**Median**	**Min. - Max.**	
CDT	Pre	6.0	0-9		4.0	0-9	0.4
	Post	7.0	0-9		5.0	1-10	0.8
VF	Pre	5.0	3-13		6.0	1-11	0.9
	Post	6.0	0-12		5.0	0-13	0.6

## DISCUSSION

The present study found no significant differences associated to the effects of the practice of strength training on memory and cognition in elderly with AD.

By contrast, some studies have found positive effects of physical activity on cognitive function in elderly with AD.^12-15^

Antunes et al.^16^ emphasized that the effects of the practice of physical activity depend on the cognitive task and type of exercise chosen.

All studies reviewed by Coelho et al.^15^ involved protocols of generalized physical activity or aerobic training. Few studies have addressed cognitive function in elderly and weight training,^43^ while fewer still have investigated the effects of such training on cognitive functions in elderly with AD.

Perrig-Chiello et al.^27^ and Lanchman et al.^44^ found no significant differences in memory in the elderly after practicing resistance training.

However, Cassilhas et al.^29^ and Liu-ambrose et al.^45^ showed positive effects of weight training on memory and cognitive functioning among elderly. Busse et al.^30^ found that older adults with memory impairment had significant improvement on tests of episodic memory after undergoing a period of resistance training.

It is important to emphasize that none of the studies mentioned above involved a sample of elderly with AD.

Thomas and Hageman^46^ found positive effects of weight training on neuromuscular function of elderly with dementia, however, the sample was heterogeneous in type of dementia and authors did not focus on the results for cognition of participants.

A review by Hurley et al.^47^ found an inverse relationship between muscle strength and AD. The systematic review conducted by Coelho et al.^15^ found that systematic physical activity can preserve or even improve cognitive function in elderly with AD.

Given the fact that the present study failed to show improvement in any of the cognitive variables tested, we suggest the implementation of resistance training with higher intensities, in order to verify whether greater intensities promote gains in cognitive levels.

This finding however, must be treated with caution since progression of cognitive decline is also related to stage of the disease. Thus, not only the protocols used but the clinical condition of elderly, still relatively preserved, could have influenced the study results.

The number of sessions in the present study may have been insufficient to generate significant results, because there were only 48 sessions of weight training, of which 15 were used to adjust the load.

Lachman et al.^44^ found that elderly people who had greater changes in load during the training program showed significant improvement in working memory. In the present study, the evolution of load achieved by all patients in the TG was observed, and the training volume was expressed by multiplying the number of sets (three sets) by the number of repetitions (20 repetitions) by the average load of all patients for each exercise.

For all periods employed, the patients showed an increase of at least 50% in the total volume of exercise. However, no significant difference in memory as assessed by the BCB was detected. Therefore, we emphasize that the number of repetitions used in this study may not have generated sufficient intensity to promote significant responses, mainly in the central levels, whereas the intensity of the present training was considered low.

First, it is important to emphasize that this low intensity of training was determined to promote greater patient safety, thus avoiding possible muscle damage.

The training protocol used in this study seems to have generated an intensity which was not sufficient to generate a satisfactory response among patients, thus being considered an exercise program of low intensity. Programs investigating higher intensities in training have tended to show different results regarding the effects of weight training and cognitive functions in elderly with AD.

Moreover, no significant difference was observed in the groups' behavior after the intervention period on the distracter tests: CDT and VF aimed at testing frontal executive functions and language.

Adherence to physical activity programs by elderly with AD remains a limitation to the research on this population. In our study, the program was widely publicized in newspapers and television and medical indications also helped patients enter the program. However, factors such as the association of AD with a clinical pathology and resistance or lack of availability of caregivers, represent barriers to the practice of physical activity by this population.

Motivation to attend the program should be constant, since it is an inherently unstable population from a behavioral point of view. In this sense, inter and intra generations should be encouraged in order to strengthen social networks. Thus, it is believed that investigation into barriers to the practice of physical activity in this population may yield further insights for future approaches.

The present study has some limitations such as the absence of a control group that did not do any kind of activity, the non-random distribution, and also the small sample size.

Accordingly, it is important to encourage further studies that investigate and manipulate the frequency, number of sets, repetitions, interval and intensity of training in order to verify other responses to resistance training in elderly with AD. Furthermore, the use of other instruments to determine cognitive functions in more depth could help to investigate the results of the training. Studies should be undertaken to verify the effects of this type of training on motor functionality of AD patients and also to ascertain how this type of training could help reduce the burden and stress of caregivers.

## References

[1] Braak E, Griffing K, Arai K (1999). Neuropathology of Alzheimer's disease: what is new since A.
Alzheimer?. Eur Arch Psychiatry Clin Neurosci.

[2] Braak H, Braak E (1997). Neuroanatomy of Alzheimer's desease. Alzheimer's Res.

[3] Braak H, Braak E (1998). Evolution of neuronal changes in the course of Alzheimer's
disease. J Neural Transm.

[4] Swerdlow RH (2007). Is aging part of Alzheimer's disease, or is Alzheimer's disease
part of aging. Neurobiol Aging.

[5] American Psychiatric Association (APA) (2000). Diagnostic and Statistical Manual of Mental Disorders.

[6] Perry RJ, Hodges JR (2000). Relationship between functional and neuropsychological
performance in early Alzheimer disease. Alzheimer Dis Assoc Disord.

[7] Aguero-Torres H, Fratiglioni l, Guo Z (1998). Dementia is the major cause of functional dependence in the
elderly: 3-year follow-up data from a population based study. Am J Public Health.

[8] Nitrini R, Caramelli P, Mansur L (2003). Neuropsicologia: das bases anatômicas à
reabilitação.

[9] Andersen CK, Wittup-Jensen KU, Lolk A (2004). Ability to perform activities of daily living is the main factor
affecting quality of life in patients with dementia. Health Qual Life Outcomes.

[10] Senanarong V, Poungvarin N, Jamjumras P (2005). Neuropsychiatric symptoms, functional impairment and executive
ability in Thai patients with Alzheimer's Disease. Int Psychogeriatr.

[11] Lanari A, Amenta F, Sivestrelli G (2007). Neurotransmitter déficits in behavioural and psychological
symptoms of Alzheimer's disease. Mech Ageing Dev.

[12] Heyn P (2003). The effect of a multisensory exercise program on engagement,
behavior, and selected physiological. Am J Alzheimer's Dis Other Demen.

[13] Liu-Ambrose T, Donaldson MG (2009). Exercise and cognition in older adults: is there a role for
resistance training programmes?. Br J Sports Med.

[14] Deslandes A, Moraes H, Ferreira C (2009). Exercise and Mental Health: Many Reasons to Move. Neurophycobiology.

[15] Coelho FGM, Santos-Galduroz RF, Gobbi S, Stella F (2009). Atividade física sistematizada e desempenho cognitivo em
idosos com demência de Alzheimer: uma revisão
sistematizada. Rev Bras Psiquiatr.

[16] Antunes HKM, Santos RF, Cassilhas R (2006). Exercício físico e função cognitiva:
uma revisão. Rev Brás Med Esporte.

[17] Larson EB, l Wang, Bowen J (2006). Exercise is associated with reduced risk for incident dementia
among persons 65 years of age and older. Ann Int Med.

[18] Kashihara K, Maruyama T, Murota M, Nakahara Y (2009). Positive Effects of Acute and Moderate Physical Exercise on
Cognitive Function. J Physiol Anthropol.

[19] Lista I, Sorrentino G (2009). Biological mechanisms of physical activity in preventing
cognitive decline. Cell Mol Neurobiol.

[20] Hernandez SSS, Coelho FGM, Gobbi S, Stella F (2010). Efeitos de um programa de atividade física nas
funções cognitivas, equilíbrio e risco de quedas em
idosos com demência de Alzheimer. Rev Bras Fisiot.

[21] Teri L, Gibbons LE, Mccurry SM (2003). Exercise plus behavioral management in patients with Alzheimer
disease: a randomized controlled trial. J Am Med Assoc.

[22] Rolland Y, Pillard F, Klapouszczak A (2007). Exercise Program for Nursing Home Residents with Alzheimer's
Disease: A 1-Year Randomized, Controlled Trial. J Am Geriatrics Soc.

[23] Arkin S (2003). Student-led exercise sessions yield significant fitness gains for
Alzheimer's patients. Am J Alzheimer's Dis Other Demen.

[24] Aman E, Thomas DR (2009). Supervised Exercise to Reduce Agitation in Severely Cognitively
Impaired Persons. J Am Med Dir Assoc.

[25] Steinberg M, Leoutsakos JS, Podewils LJ, Lyketsos CG (2009). Evaluation of a home-based exercise program in the treatment of
Alzheimer's disease: The Maximizing Independence in Dementia (MIND)
study. Int J Geriatr Psychiatry.

[26] Arcoverde C, Deslandes A, Rangel A (2008). Role of physical activity on the maintenance of cognition and
activities of daily living in elderly with Alzheimer's
disease. Arq Neuropsiquiatr.

[27] Perrig-Chiello P, Perrig WJ, Ehrsam R, Staehelin HB, Krings F (1998). The effects of resistance training on well-being and memory in
elderly volunteers. Age Ageing.

[28] Özkaya GY, Aydin H, Toraman FN, Kizilay F, Ozdemir O, Cetinkaya V (2005). Effect of strength and endurance training on cognition in older
people. J Sports Sci Med.

[29] Cassilhas RC, Viana VA, Grassmann V (2007). The Impact f Resistance Exercise on the Cognitive Function of the
Elderly. Med Sci Sport Exerc.

[30] Busse AL, Jacob-Filho W, Magaldi RM (2008). Effects of Resistance Training Exercise on Cognitive Performance
in Elderly Individuals with Memory Impairment: Results of a Controlled
Trial. Einstein.

[31] Garuffi M, Gobbi S, Hernandez SSS (2011). Atividade física para promoção da
saúde de idosos com doença de Alzheimer e seus
cuidadores. Rev Bras Ativ Fís Saúde.

[32] Morris J (1993). The Clinical Dementia Rating (CDR): current version and scoring
rules. Neurology.

[33] Montaño MBMM, Ramos LR (2005). Validade da versão em português da Clinical
Dementia Rating (CDR). Rev Saúde Pública.

[34] Voorrips L, Ravelli A, Dongelmans P, Deurenberg P, Van Staveren W (1991). A physical activity questionnaire for elderly. Med Sci Sports Exerc.

[35] Folstein MF, Folstein SE, Mchugh PR (1975). Mini-mental state. A practical method for grading the cognitive
patients for the clinician. J Psychiatry Res.

[36] Brucki SMD, Nitrini R, Caramelli P, Bertolucci PHF, Okamoto JH (2003). Suggestions for the utilization of the mini-mental state
examination in Brazil. Arq Neuropsiquiatr.

[37] Nitrini R, Lefèvre BH, Mathias SC (1994). Testes neuropsicológicos de aplicação
simples para o diagnóstico de demências. Arq Neuropsiquiatr.

[38] Nitrini R, Caramelli P, Porto CS (2007). Brief cognitive battery in the diagnosis of mild Alzheimer's
disease in subjects with medium and high levels of education. Dement Neuropsychol.

[39] Christofoletti G, Oliani MM, Stella F, Gobbi S, Gobbi LTB (2007). Influence of scholarity on cognitive screening test in elderly
people. Dement Neuropsychol.

[40] Sunderland T, Hill JL, Mellow AM (1989). Clock drawing in Alzheimer's disease. A novel measure of dementia
severity. J Am Geriatrics Soci.

[41] Lezak MD (1995). Neuropsychological Assessment.

[42] Garuffi M, Costa JLR, Hernández SSS (2012). Effects of resistance training on the performance of activities
of daily living in patients with Alzheimer's disease. Geriatrics and Gerontology International.

[43] Busse AL, Gil G, Santarém JM, Filho WJ (2009). Physical activity and cognition in the elderly. Dement Neuropsychol.

[44] Lachman ME, Neupert SD, Bertrand R, Jette AM (2006). The effects of strength training on memory in older
adults. J Aging Physl Act.

[45] Liu-Ambrose T, Donaldson MG, Ahamed Y (2008). Otago home-based strength and balance retraining improves
executive functioning in older fallers: a randomized controlled
trial. J Am Geriatr Soc.

[46] Thomas VS, Hageman PA (2003). Can neuromuscular strength and function in people with dementia
be rehabilitated using resistance-exercise training? Results from a
preliminary intervention study. J Gerontol Med Sci.

[47] Hurley BF, Hanson ED, Sheaff AK (2011). Strength training as a countermeasure to aging muscle and chronic
disease. Sports Med.

